# Fast inactivation of SARS-CoV-2 by UV-C and ozone exposure on different materials

**DOI:** 10.1080/22221751.2021.1872354

**Published:** 2021-02-02

**Authors:** Elena Criscuolo, Roberta A. Diotti, Roberto Ferrarese, Cesare Alippi, Gabriele Viscardi, Carlo Signorelli, Nicasio Mancini, Massimo Clementi, Nicola Clementi

**Affiliations:** a Laboratory of Microbiology and Virology, Vita-Salute San Raffaele University, Milan, Italy; b Dipartimento di Elettronica e Informazione, WEMSY Lab, Politecnico di Milano, Italy; c School of Medicine, Vita-Salute San Raffaele University, Milan, Italy; d Laboratory of Microbiology and Virology, IRCCS San Raffaele Scientific Institute, Milan, Italy

**Keywords:** Ozone, UV-C, SARS-CoV-2, inactivation, contact transmission

## Abstract

The extremely rapid spread of the SARS-CoV-2 has already resulted in more than 1 million reported deaths of coronavirus disease 2019 (COVID-19). The ability of infectious particles to persist on environmental surfaces is potentially considered a factor for viral spreading. Therefore, limiting viral diffusion in public environments should be achieved with correct disinfection of objects, tissues, and clothes. This study proves how two widespread disinfection systems, short-wavelength ultraviolet light (UV-C) and ozone (O3), are active in vitro on different commonly used materials. The development of devices equipped with UV-C, or ozone generators, may prevent the virus from spreading in public places.

The World Health Organization (WHO) declared SARS- CoV-2 a pandemic on 11th March 2020. As of the 3rd November 2020, there have been over 46.8 million confirmed COVID-19 cases and more than 1 million reported deaths [1]. The main transmission route of this virus appears to be via aerosols [2], and another suggested mode involves fomites [3]. The persistence of SARS-CoV-2 on environmental surfaces is potentially considered a critical factor for viral spreading, although there are conflicting reports on the maintenance of infectivity of SARS-CoV-2 on different surfaces [4,5]. For this reason, the correct disinfection of surfaces, tissues and clothes may play an important role in limiting the viral diffusion through hospitals, hotels, nursing homes, and housing. Virucidal activity on SARS-CoV-2 of different systems such as alcohol-based disinfectant, heat, chemicals, and, recently, the role of DUV-LED on a plastic surface, was investigated [6]. Overall, these data reveal that viral infectivity on surfaces is influenced by many factors including the viral load absorbed on the environmental surfaces. To better translate data generated in laboratory conditions to everyday life, the viral load, used in the different experimental protocols, should be similar to that possibly present on contaminated surfaces. Published data equate a virus amount of 10^5^ TCID_50_/mL to a cycle threshold (Ct) value ranging from 20 to 22, depending on the diagnostic platforms adopted, for SARS-CoV-2 Real-Time PCR [4] and other studies have reported COVID-19 patients with a very high viral load on nasopharyngeal swabs corresponding to Ct values ranging from 13–15 [7]. Thus, a virus concentration of 1.5 × 10^6^ TCID_50_/ mL can represent a reasonable amount of virus that may be deposited on a surface to evaluate experimentally the virucidal activity of sterilizing procedures. No peer-reviewed report predicts the virus concentration from the droplets of sneezing or coughing, but a preprint manuscript by Schijven *et al.* describes that the range of observed SARS-CoV-2 concentration in swab samples of 10^2^–10^11^ RNA copies/mL led to the calculated range of viral concentrations in the air (from 10^−4^–10^2^ per liter of air), that encompass the values of observed airborne SARS-CoV-2 concentrations in hospital rooms with SARS-CoV-2 patients [8].

Here, two widespread disinfection systems (short-wavelength ultraviolet light (UV-C) and ozone (O_3_)) are investigated for their efficacy. The 40 W germicidal lamp, wavelength 254 nm (UV-C) is commonly adopted for the sterilization of stainless-steel worktop in the laminar flow cabinets; while, ozone, a highly oxidizing gas, is normally used for the disinfection of municipal water, foods, and surfaces [9]. Ozone is highly corrosive to equipment and is lethal to humans with prolonged exposure at concentrations above 4 ppm. The U.S. Food and Drug Administration (FDA)’s maximum allowed ozone concentration in the air for residential areas is 0.05 ppm ozone by volume. For work environments, the U.S. Department of Labor’s Occupational Safety & Health Administration (OSHA)’s Permissible Exposure Limit for General, Construction and Maritime Industry is a 0.1 ppm time-weighted average (0.2 mg/m^3^). The application of ozone for direct contact on foods was not approved as Generally Recognized As Safe (GRAS) by the FDA until June 2001 under the FDA Final Rule 21 CFR Part 173.336. Later that year the U.S. Dept. of Agriculture’s Food Safety Inspection Service approved ozone for use on meat and poultry products. Aqueous ozone has been used to treat meat at 0.2 ppm ozone for up to 60 min with storage up to 24 days. The FDA further recognized ozone as a Good Manufacturing Practice for bottled water, with a minimum treatment of 0.1 mg/L. In clean, potable water free of organic debris and soil particulates, ozone is a highly effective sanitizer at concentrations of 0.5–2 ppm (1 mg/L = 1 ppm) [10].

To the best of our knowledge, there are no studies investigating on the virucidal activity of both UV-C and O_3_ against clinical isolates of SARS-CoV-2 absorbed on commonly used materials. Other groups described fully viral inactivation by UV-C treatment achieved in a short time (20 s to 9 min), but with some important differences from our data. Firstly, effective treatments shorter than 1 min were tested on two logarithms-less infectious viral stock than the one used in the present study [6]. Besides, 9 min-treatment proved to be sufficient for SARS-CoV-2 inactivation when UV-C was combined with UV-A [11]. Importantly, in both experimental settings, the virus was adsorbed to slides or plastic plates, and only 2-3 cm away from the light sources. As our results describe UV-C treatment of virus adsorbed on different materials and at a distance of 20 cm from the light source, our considerations might be translated in an easier way for fast feasible surface treatment.

Therefore, we designed two experimental settings aimed at evaluating and comparing the sterilizing capability of these two systems on high dose of SARS-CoV-2 adsorbed on different materials.

A clinical isolate hCoV-19/Italy/UniSR1/2020 (GISAID accession ID: EPI_ISL_413489) was isolated and propagated in Vero E6 cells, the supernatant was collected 48 hpi and stored at −80°C in Dulbecco's Modified Eagle Medium (DMEM) supplemented with non-essential amino acids (NEAA, 1x), penicillin/streptomycin (P/S, 100 U/mL), HEPES buffer (10 mM) and 2% (v/v) Fetal bovine serum (FBS), as previously described [12] (Appendix). Then, viral titer was determined by 50% tissue culture infective dose (TCID_50_) and plaque assay (Plaque forming units, PFU).

In the first experimental setting aimed to evaluate UV-C activity, aliquots of viral stock (50 μL, 1.5 × 10^6^ TCID_50_/mL, equal to 8.2 × 10^5^ PFU/mL) were placed in a 24-well plate in ice to counteract irradiation-derived heating of the sample, and irradiated with approximately 1.8 mW/cm^2^ at a work distance of 20 cm for a range of times (15, 30 and 45 min, corresponding to 1.62, 3.24 and 4.86 J/cm^2^, respectively [13]). Then, 500 μL of medium without FBS were added to wells, collected after 5 min, and stored at −80°C to be back titrated on Vero E6 cells to evaluate if the treatment eliminated all the infectious viral particles. Briefly, Vero E6 cells (4 × 10^5^ cells/mL) were seeded into 96-wells plates and infected with base 10 dilutions of the collected medium, each condition tested in triplicate. After 1 h of adsorption at 37°C, complete medium was added to cells after a PBS 1x wash. After 72 h, cells were observed to evaluate CPE. TCID_50_/mL was calculated according to the Reed–Muench method. The back titration was preferred to Real-Time PCR because the detection of viral genomes is not suitable to distinguish between infectious and non-infectious particles[14]. The infectious titer reduction rates were calculated as (1–1/10^log10 (N0/Nt)^) × 100 (%), where Nt is the titer of the UV-irradiated sample, and N0 is the titer of the sample without irradiation[6]. Results showed that 15 min of irradiation were sufficient to reduce the viral titer of >99.9% (30 and 45 min resulted in >99.9% reduction of infectious titers).

Thus, the virus inactivation ability of UV-C on different surfaces was tested with an irradiation time of 15 min. We selected six types of materials of common use: glass (13 mm round glass coverslips), plastic (cap of 0.2 mL PCR tube), gauze (sterile gauze pad), wood (sterile wood tongue depressor), fleece, and wool (both sterilized by bleaching). Fabric and wood samples were prepared by cutting 0.5 cm x 0.5 cm swatches. The samples were put into 24-wells on ice, irradiated, and the virus was eluted and collected at −80°C for back titration. The infectious titer reduction rates showed a complete inactivation (>99.9%) on glass, plastic, and gauze, and a less marked virucidal effect on the other two fabrics (90% for fleece, 94.4% for wool) (Tab. S1). The irradiation used was not sufficient to reduce virus titer on the wood sample (0%).

In the experimental setting aimed at evaluating the ozone activity, the Ozonext Defender 10 (Cea S.p.A., Lecco, Italy) was adapted to be used inside a system composed of a plexiglass chamber containing the contaminated samples connected to an ozone detector, to monitor gas concentration (part per million, ppm) throughout all the experimental sessions. Firstly, the six materials were placed into a 24-well plate and tested using 0.2 ppm, a gas concentration non-toxic to humans [10], for 2 h, as this time point replicates the longest treatment which can be selected on commercially available ozone generators (Figure 1A). This would allow, possibly, the sanitization of places without closing access to the public. Results showed that complete disinfection was obtained only on fleece sample (>99.9%), while a less marked reduction was observed on the other materials (96.8% on gauze, 93.3% on wood, 90% on glass), with the worst data was observed on plastic (82.2%) (Tab. S2). Unexpected toxicity was observed on back titration experiments on Vero E6 for wool specimens, maybe due to chemical pre-experimental sterilization. Thus, it was not included in the subsequent experiment.

**Figure 1.**
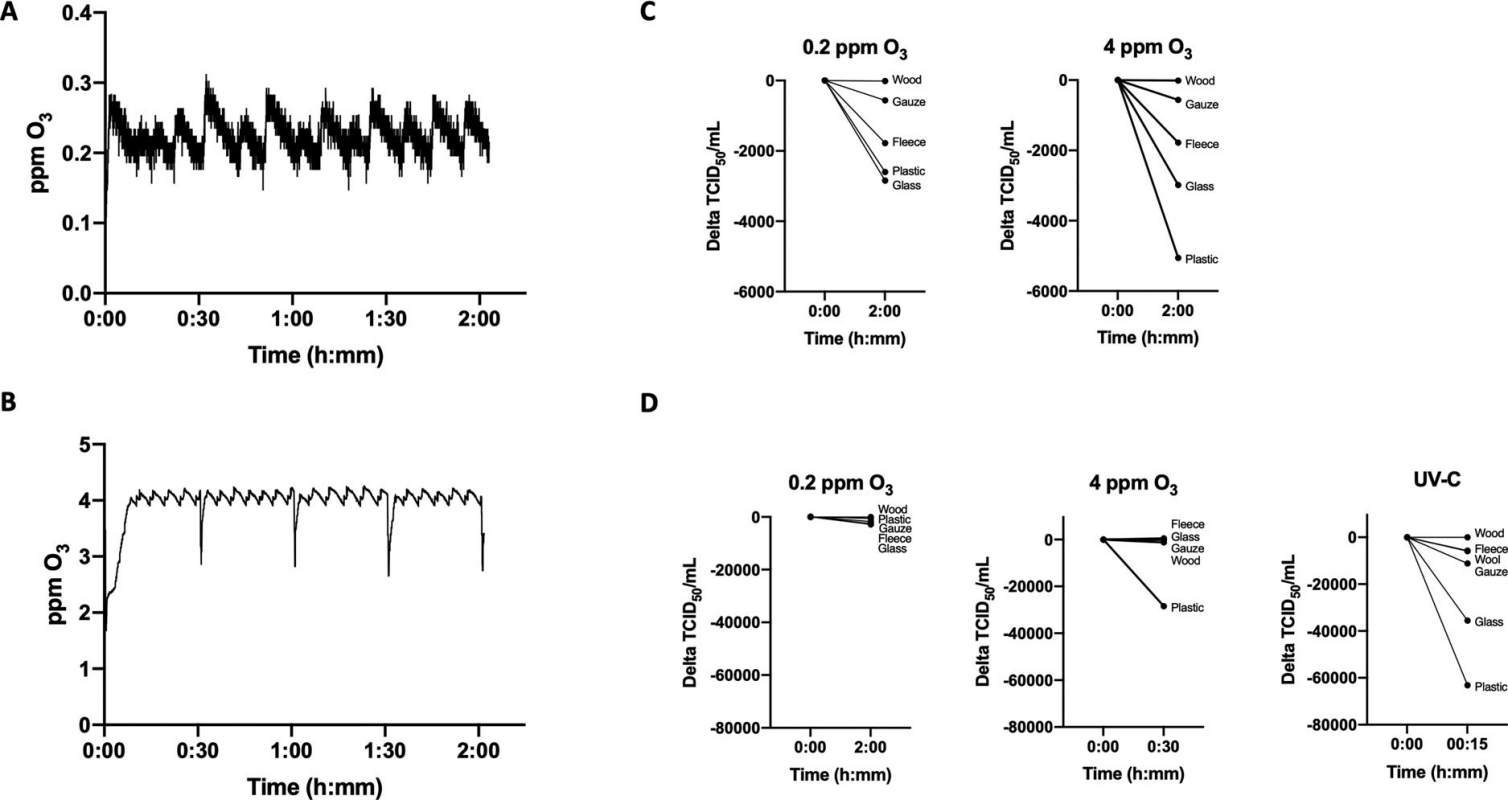
Monitoring of O_3_ concentration. The gas concentration in the plexiglass chamber was monitored during the experiments. A) The 0.2 ppm concentration was tested for a single two-hour time point; despite being very low, the system managed to keep the oscillations from the desired concentration to a minimum. B) The effects of a higher concentration (4 ppm) were tested at 4 time-points, and the peaks in the graph correspond to the opening of the plexiglass chamber, demonstrating how they did not affect the concentration of the gas inside. Comparison of SARS-CoV-2 titer reduction on different materials, using UV-C and ozone exposure. C) The effect of 0.2 and 4 ppm on virus titers after 2 h of exposure. D) The effect of treatment with low and high concentrated ozone is compared to UV-C exposure at their shorter tested time points (2 h, 30 and 15 min, respectively). Delta TCID_50_/mL was calculated as the difference between titers obtained from treated and untreated materials.

To investigate a possible use of ozone for quick sanitization of closed places in the absence of people, a higher ozone concentration (4 ppm) was evaluated at different times of exposure: 30, 60, 90, and 120 min (Figure 1B). The results of titer reduction experiments showed that the effect on glass and gauze was maximum (a 98.2% and 99.8% viral titer reduction, respectively) after 90 min of exposure, while 120 min are required to sanitize fleece almost completely (99.8%), and plastic of 90% (Tab. S3). Reduction of infectious titers, reported in tables S2 and S3, describes the reduction of infectious capability of the virus adsorbed on different materials over the time. Results are obtained by comparison between O_3_-treated virus and untreated virus, left at room temperature up to 120 min. Data showed how also untreated virus stocks were affected by a lowering of infectivity over time, at different time points. This observation does not disagree with literature data on SARS-CoV-2 persistence [5,15] . Finally, in our experimental conditions wood cannot be disinfected better than 93.3%, a result already obtained after a 30 min treatment.

This study demonstrated for the first time the inactivation of SARS-CoV-2 on different materials under UV-C irradiation and ozone exposure. Unexpectedly, the higher ozone concentration tested in our experiments did not result in better decontamination of surfaces compared to lower one, except for plastic (Figure 1C). However, when comparing both O_3_ concentration to UV-C quick treatments, our data showed that irradiation was more effective for all tested conditions (Figure 1D). The range of the difference between titers obtained from treated and untreated materials (Delta TCID_50_/mL) resulted in extreme differences between O_3_ and UV-C treatments (3,000 and 70,000 maximum with low dose O_3_ and UV-C respectively), making the light treatment 1 log more effective in SARS-CoV-2 decontamination of certain materials (i.e. plastic). Unfortunately, it was not possible to test high O_3_ concentration on shorter time points because, as shown in Figure 1B, the system required time to reach the desired gas concentration after the opening of the plexiglass chamber to collect the specimens (lowering of gas pressure: - 1.369 ppm ± 0.196; restored in approximately 2 min). Thus, the analysis of subsequent time points with intervals of less than 30 min (i.e. 15 min) would have affected the reliability of our results. For the same reason, it was not possible to test low O_3_ concentration treatment for shorter time points.

Interestingly, wood cannot be fully decontaminated with any protocol, probably for its porous nature that may offer physical shelter to virus particles, but also trapping them and preventing their elution. Fleece proved difficult to be fully decontaminated using short-time treatments, but UV-C allowed to reach 90% of reduction.

We confirmed for the first time that the rapid antiviral activity of UV-C observed by other groups on slides or plastic plates at a work distance of 2-3 cm from the light source are reproducible on fabric and materials specimens 20 cm away from the UV-C lamp. In detail, we showed that treatment as rapid as 15 min is sufficient to completely inactivate any viral particle present on different materials, making our considerations easily applicable for feasible surface treatment. Moreover, our results show that different types and durations of ozone exposure led to a significant reduction of viral titer on the tested materials, providing useful data toward securing public environments. A rapid treatment using 4 ppm O_3_ for 30 min led to a reduction of the viral titers above 90% for almost all tested materials. As expected, lower gas concentrations non-toxic to humans required four times as much time to achieve the same result. The development of devices equipped with UV-C, or the use of ozone generators, are expected to limit the virus from spreading through contaminated objects and surfaces in highly frequented public places, such as nosocomial areas, where it is more difficult to apply thorough surface hygiene.

## Supplementary Material

EMI_Appendix_1.docx
