# Supplementary material for: Fast inactivation of SARS-CoV-2 by UV-C and ozone exposure on different materials
Source: Emerg Microbes Infect. 2021 Feb 2;10(1):206–9. doi: 10.1080/22221751.2021.1872354 (PMC7872580; doi:10.1080/22221751.2021.1872354)
Supplement: EMI_Appendix_1.docx [file TEMI_A_1872354_SM1231.docx]

**Supplementary Materials for**

**Fast inactivation of SARS-CoV-2 by UV-C and ozone exposure on different materials**

**Materials and methods**

***Cells and Virus***

Vero E6 (Vero C1008, clone E6 - CRL-1586; ATCC) cells were cultured in Dulbecco's Modified Eagle Medium (DMEM) supplemented with non-essential amino acids (NEAA, 1x), penicillin/streptomycin (P/S, 100 U/mL), HEPES buffer (10 mM) and 10% (v/v) Fetal bovine serum (FBS). A clinical isolate hCoV-19/Italy/UniSR1/2020 (GISAID accession ID: EPI_ISL_413489) was isolated and propagated in Vero E6 cells, and viral titer was determined by 50% tissue culture infective dose (TCID_50_) and plaque assay for confirming the obtained titer. All the infection experiments were performed in a biosafety level-3 (BLS-3) laboratory of Microbiology and Virology at Vita-Salute San Raffaele University, Milan, Italy.

***Virus Isolation***

An aliquot (0.8 mL) of the transport medium of the nasopharyngeal swab (COPAN's kit UTM® universal viral transport medium - COPAN) of a mildly symptomatic SARS-CoV-2 infected patient was mixed with an equal volume of DMEM without FBS and supplemented with double concentration of P/S and Amphotericin B, as previously described [1]. The mixture was added to 80% confluent Vero E6 cells monolayer seeded into a 25 cm^2^ tissue culture flask. After 1 h adsorption at 37°C, 3 mL of DMEM supplemented with 2% FBS and Amphotericin B were added. 24 hours post-infection (hpi) another 2 mL of DMEM supplemented with 2% FBS and Amphotericin B were added. Live images were acquired (Olympus CKX41 inverted phase-contrast microscopy) daily for evidence of cytopathic effects (CPE), and aliquots were collected for viral RNA extraction and In-house one-step Real-Time PCR assay [2]. Five days post-infection (dpi) cells and supernatant were collected, aliquoted, and stored at −80°C (P1). For secondary (P2) virus stock, Vero E6 cells seeded into 25 cm^2^ tissue culture flasks were infected with 0.5 mL of P1 stored aliquot, and infected cells and supernatant were collected 48 hpi and stored at −80°C. For tertiary (P3) virus stock, Vero E6 cells seeded into 75 cm2 tissue culture flasks were infected with 1.5 mL of P2 stored aliquot and prepared as above described.

***Virus Titration***

P3 virus stocks were titrated using both Plaque Reduction Assay (PRA, PFU/mL) and Endpoint Dilutions Assay (EDA, TCID_50_/mL). For PRA, confluent monolayers of Vero E6 cells were infected with 10-fold-dilutions of virus stock. After 1 h of adsorption at 37°C, the cell-free virus was removed. Cells were then incubated for 46 h in DMEM containing 2% FBS and 0.5% agarose. Cells were fixed and stained, and viral plaques were counted. For EDA, Vero E6 cells (4 × 10^5^ cells/mL) were seeded into 96 wells plates and infected with base 10 dilutions of virus stock. After 1 h of adsorption at 37°C, the cell-free virus was removed, and complete medium was added to cells. After 48 h, cells were observed to evaluate CPE. TCID_50_/mL was calculated according to the Reed–Muench method.

***Sequence Analysis***

Viral genome from supernatant infected cells was extracted using QIAamp Viral RNA Mini Kit following manufacturers' instructions. Reverse transcription and subsequent amplification were performed using random hexamer primers. The amplicons were sequenced on the Illumina MiSeq NGS platform (Illumina, San Diego, CA, USA). Amplicon purification and quantification were performed by Agencourt AMPure XP (Beckman Coulter, Villepinte, France) and Qubit dsDNA Assay Kit (ThermoFisher Scientific, Waltham, MA, USA), respectively. Library preparation was performed by using the Nextera XT DNA Library Prep Kit (Illumina, San Diego, CA, USA). The library generated was then diluted and sequenced with MiSeq Reagent Kit v2 (300-cycles) (Illumina, San Diego, CA, USA) on the MiSeq platform. The quality of raw sequences obtained from MiSeq run was first checked using FastQC (v 0.11.5) (Babraham Bioinformatics). The reads were aligned on reference sequence (GISAID accession ID: EPI_ISL_412973) using BWA-mem and rescued using Samtools alignment/Map (v 1.9) and bamtoFastq. Finally, the contigs were generated using SPAdes (v 3.12.0).

***UV-C antiviral activity evaluation at different time points***

Aliquots of viral stock (50 μL, 1.5 x 10^6^ TCID_50_/mL, equal to 8.2 x 10^5^ PFU/mL) were placed in a 24-well plate in ice, and irradiated with approximately 1.8 mW/cm^2^ at a work distance of 20 cm for a range of times (15, 30 and 45 minutes, corresponding to 1.62, 3.24 and 4.86 J/cm^2^, respectively [3]. Then, 500 μL of medium without FBS were added to wells, collected after 5 minutes, and stored at -80°C to be back titrated on Vero E6 cells. Briefly, Vero E6 cells (4 × 10^5^ cells/mL) were seeded into 96-wells plates and infected with base 10 dilutions of collected medium, each condition tested in triplicate. After 1 h of adsorption at 37°C, complete medium was added to cells after a PBS 1x wash. After 72 h, cells were observed for CPE evaluation, and TCID_50_/mL was calculated as described. The infectious titer reduction rates were calculated as (1–1/10log10 (N0/Nt)) × 100 (%), where Nt is the titer of the UV-irradiated sample, and N0 is the titer of the sample without irradiation [4].

***UV-C antiviral activity evaluation on different materials***

Six types of materials of common use were selected: glass (13 mm round glass coverslips), plastic (cap of 0.2 mL PCR tube), gauze (sterile gauze pad), wood (sterile wood tongue depressor), fleece, and wool (both sterilized by bleaching). Fabric and wood samples were prepared by cutting 0.5 cm x 0.5 cm swatches. The samples were put into 24-wells on ice with aliquots of viral stock (50 μL, 1.5 x 10^6^ TCID_50_/mL, equal to 8.2 x 10^5^ PFU/mL) and irradiated for 15 minutes. Then the virus was eluted and collected at -80°C to perform a back titration the following day as above described.

***Ozone*** ***antiviral activity evaluation on different materials***

The Ozonext Defender 10 (Cea S.p.A., Lecco, Italy) was adapted to be used inside a system composed of a plexiglass chamber containing the contaminated samples and connected to an ozone detector to monitor gas concentration (part per million, ppm). The selected six materials were placed into a 24-well plate, contaminated with 50 μL of viral stock (1.5 x 10^6^ TCID_50_/mL, equal to 8.2 x 10^5^ PFU/mL), and tested using 0.2 ppm for 2 hours, or 4 ppm for different times of exposure (30, 60, 90, and 120 minutes). Then the virus was eluted and collected at -80°C for back titration as above described.

**Supplemental tables**

**Table S1**. Titers reduction after 15 minutes of UV-C irradiation on different materials.

| **Material** | **Infectious titer reduction rate^a^ (%)** |
| --- | --- |
| Glass | >99.9 |
| Plastic | >99.9 |
| Gauze | >99.9 |
| Wood | 0.0 |
| Fleece | 90.0 |
| Wool | 94.4 |

^a^ 1–1/10^log10 (N0/Nt)^) × 100

**Table S2**. Titers reduction after disinfection treatment with O_3_ at low concentration for 2 hours.

| **Material** | **Infectious titer reduction rate^a^ (%)** |
| --- | --- |
| Glass | 90.0 |
| Plastic | 82.2 |
| Gauze | 96.8 |
| Wood | 93.3 |
| Fleece | >99.9 |

^a^ 1–1/10^log10 (N0/Nt)^) × 100

**Table S3**. Titers reduction after disinfection treatment with O_3_ at high concentration.

| **Materials** | **Infectious titer reduction rate^a^ (%)** | | | |  |
| --- | --- | --- | --- | --- | --- |
|  | **00:30** | **1:00** | **1:30** | **2:00** | **(h:mm)** |
| Glass | 0.0 | 93.2 | 98.2 | 94.4 |  |
| Plastic | 90 | 68.4 | 68.3 | 90.0 |  |
| Gauze | 68.4 | 99.2 | 99.8 | 99.8 |  |
| Wood | 93.3 | 93.3 | 93.3 | 0.0* |  |
| Fleece | 0.0 | 96.8 | 94.4 | 99.7 |  |

^a^ 1–1/10^log10 (N0/Nt)^) × 100. The reported values refer to the direct comparison between treated and untreated (left at room temperature, without O_3_ exposure) materials, evaluated at the different time points. * No infectious titer was detected with back titration in both treated and untreated collected specimens.

**References**

[1] N. Clementi, E. Criscuolo, R.A. Diotti, R. Ferrarese, M. Castelli, L. Dagna, et al., Combined Prophylactic and Therapeutic Use Maximizes Hydroxychloroquine Anti-SARS-CoV-2 Effects in vitro, Front. Microbiol. 11 (2020) 1704. doi:10.3389/fmicb.2020.01704.

[2] J.F.-W. Chan, S. Yuan, K.-H. Kok, K.K.-W. To, H. Chu, J. Yang, et al., A familial cluster of pneumonia associated with the 2019 novel coronavirus indicating person-to-person transmission: a study of a family cluster, Lancet. 395 (2020) 514–523. doi:10.1016/S0140-6736(20)30154-9.

[3] R.M. Gilbert, M.J. Donzanti, D.J. Minahan, J. Shirazi, C.L. Hatem, B. Hayward-Piatkovskyi, et al., Mask Reuse in the COVID-19 Pandemic: Creating an Inexpensive and Scalable Ultraviolet System for Filtering Facepiece Respirator Decontamination, Glob Health Sci Pract. 8 (2020) 582–595. doi:10.9745/GHSP-D-20-00218.

[4] H. Inagaki, A. Saito, H. Sugiyama, T. Okabayashi, S. Fujimoto, Rapid inactivation of SARS-CoV-2 with deep-UV LED irradiation, Emerging Microbes & Infections. 9 (2020) 1744–1747. doi:10.1080/22221751.2020.1796529.
